# Regulatory Mechanisms of the Ihh/PTHrP Signaling Pathway in Fibrochondrocytes in Entheses of Pig Achilles Tendon

**DOI:** 10.1155/2016/8235172

**Published:** 2016-11-22

**Authors:** Xuesong Han, Yanfeng Zhuang, Zhihong Zhang, Lin Guo, Wanming Wang

**Affiliations:** ^1^Department of Orthopedics, Fuzhou General Hospital of Nanjing Command, PLA, Fuzhou 350025, China; ^2^Center of Joint Surgery, Southwest Hospital, The Third Military Medical University, Chongqing 400038, China

## Abstract

This study is aimed at exploring the effect of stress stimulation on the proliferation and differentiation of fibrochondrocytes in entheses mediated via the Indian hedgehog (Ihh)/parathyroid hormone-related protein (PTHrP) signaling pathway. Differential stress stimulation on fibrochondrocytes in entheses was imposed. Gene expression and protein levels of signaling molecules including collagen type I (Col I), Col II, Col X, Ihh, and PTHrP in the cytoplasm of fibrochondrocytes were detected. Ihh signal blocking group was set up using Ihh signaling pathway-specific blocking agent cyclopamine. PTHrP enhancement group was set up using PTHrP reagent. Ihh/PTHrP double intervention group, as well as control group, was included to study the regulatory mechanisms of the Ihh/PTHrP signaling pathway in fibrochondrocytes. Under low cyclic stress tensile (CTS), PTHrP, Col I, and Col II gene expression and protein synthesis increased. Under high CTS, Ihh and Col X gene expression and protein synthesis increased. Blocking Ihh signaling with cyclopamine resulted in reduced PTHrP gene expression and protein synthesis and increased Col X gene expression and protein synthesis. Ihh and PTHrP coregulate fibrochondrocyte proliferation and differentiation in entheses through negative feedback regulation. Fibrochondrocyte is affected by the CTS. This phenomenon is regulated by stress stimulation through the Ihh/PTHrP signaling pathway.

## 1. Introduction

Entheses damage and degeneration are common motor system diseases, with no ideal treatments at present [1]. The organizational structure of entheses has four layers, of which the fibrous cartilaginous layer plays an important role in entheses development and degeneration [2]. The development of entheses is associated with the proliferation and differentiation of fibrochondrocytes.

Stress stimulation is a major influencing factor for entheses development and degeneration [3–7]. Stress stimulation affects the balance of extracellular matrix synthesis and decomposition of chondrocytes, and hence changes the metabolism of articular chondrocytes. Multiple cytokines participate in the proliferation and differentiation of chondrocytes in entheses. Among these, stress-related protein parathyroid hormone-related protein (PTHrP) is known to promote chondrocyte proliferation and cartilage layer development [8–10]. Indian hedgehog (Ihh) is an upstream molecule of PTHrP [11]. Ihh is an important regulatory molecule during chondrocyte proliferation and differentiation. PTHrP is an indispensable limiting regulatory factor for chondrocyte proliferation and differentiation and controlled mainly by its upstream secretory protein Ihh. Ihh and PTHrP signals together maintain the dynamic equilibrium of chondrocyte development and metabolism [12, 13]. However, how stress stimulation mediates Ihh/PTHrP signaling pathway to regulate chondrocyte proliferation and differentiation in entheses is largely unknown.

Therefore, it is worthwhile to study how stress stimulation mediates Ihh/PTHrP signaling pathway to regulate chondrocyte proliferation and differentiation in entheses, which has an extensive application value in clinic.

## 2. Materials and Methods

### 2.1. Reagents

#### 2.1.1. Cyclopamine (Final Concentration = 10 *µ*M)

Cyclopamine hydrate was procured from Sigma (C4116-1MG, lot # 021M4704V, P code: 11211).

Cyclopamine was centrifuged at 1000 rpm for 5 min, and 200 *µ*L of dimethyl sulfoxide was added to dissolve cyclopamine completely. Then, 240 mL of Dulbecco's modified Eagle's medium (DMEM) with 10% fetal bovine serum (FBS) and 1% antibiotics was added to rinse the reagent bottle 5 times.

#### 2.1.2. PTHrP (Final Concentration = 10 nM)

Human PTHrP was purchased from Peprotech (cat# 100-09 50 *µ*g, lot# 1002267, L122). PTHrP was centrifuged at 1000 rpm for 5 min. Then, 5 mL of DMEM with 10% FBS and 1% antibiotics was added to wash the reagent bottle five times and stored in 10 mL centrifuge tubes. PTHrP (1 mL) was added into 99 mL of DMEM (with 10% FBS and 1% antibiotics). The remaining reagents in the centrifuge tubes were stored at 8°C.

### 2.2. Antibodies

*PTHrP.* 1 : 1000 dilution. The primary antibody was from rabbits, and the secondary antibody was goat anti-rabbit (1 : 1000 dilution).

*Ihh.* 1 : 1000 dilution. The primary antibody was from rabbits, and the secondary antibody was goat anti-rabbit (1 : 1000 dilution).

*Collagen 1A I (Col I).* 1 : 500 dilution. Collagen 1A I was from goat, and the secondary antibody was rabbit anti-goat (1 : 1000 dilution).

*Collagen II.* 1 : 1000 dilution. Collagen II was from mouse, and the secondary antibody was goat anti-mouse (1 : 1000 dilution).

*Collagen X.* (1 : 1000 dilution). Collagen X was from mouse, and the secondary antibody was goat anti-mouse (1 : 1000 dilution).

*Actin.* 1 : 1000 dilution. Actin was from mouse, and the secondary antibody was goat anti-mouse (1 : 1000 dilution).

All secondary antibodies were from Zhongshan Co. (Beijing, China).

### 2.3. Animals

We used two Guizhou miniature pigs (Experimental Animal Centre of the Third Military Medical University, Chongqing, China), weighing 18 kg and aged 5 months. Animals were treated according to the NIH Guide for the Care and Use of Laboratory Animals (NIH 2011). The experiments were approved by the Third Military Medical University Committee for Animal Experimentation.

### 2.4. Cell Culture

The fibrochondrocytes were collected by two-step enzyme digestion [14]. We separated and cut the calcified fibrocartilaginous layer and Fibrocartilage tissue was chopped into pieces then placed in a digestion chamber. After incubation with 0.2% trypsin for 2 h and 0.2% collagenase for 18 h (GIBCO, Invitrogen Inc., Carlsbad, CA, USA), fibrocartilaginous cells were centrifuged, and the pellet was resuspended in Dulbecco's modified eagle serum (DMEM) (GIBCO, Invitrogen Inc., Carlsbad, CA, USA) supplemented with 10% fetal bovine serum (FBS) (GIBCO, Invitrogen Inc., Carlsbad, CA, USA) and 1% penicillin/streptomycin (GIBCO, Invitrogen Inc., Carlsbad, CA, USA). Cells were seeded into a T-25 vented flask, were grown to 80–90% confluence, and were used at the third passage. Fibrochondrocytes at the third passage were characterized using hematoxylin-eosin (HE) staining, alcian blue staining (indicative of glycosaminoglycan), and types I and II collagen immunohistochemistry. After identification, the culture cells were passaged to the second generation and plated at a density of 2 × 10^5^ in a Bioflex six-well plate coated with Col I. The cells were incubated in a 37°C constant-temperature incubator with 5% CO_2_. The media was changed the next day. The cells were cultured until the six-well plate was overspread with 85%–90% cells.

### 2.5. Experimental Parameters

*Control Group.* Control, DMEM + 10% FBS + 1% double-antibiotics, 10 mL.

*Ihh Signal Blocking Group.* 10 *µ*M cyclopamine in DMEM + 10% FBS + 1% double-antibiotics, 10 mL.

*PTHrP Signal Enhancement Group.* 10 nM PTHrP in DMEM + 10% FBS + 1% double-antibiotics, 10 mL.

*Double-Intervention Group.* 10 *µ*M cyclopamine and 10 nM PTHrP in DMEM + 10% FBS + 1% double-antibiotics, 10 mL.

### 2.6. Detection Index

*RT-PCR Targets.* Ihh, PTHrP, Col I, Col II, and Col X.

*Western Blot Targets.* Ihh, PTHrP, Col I, Col II, and Col X.

### 2.7. Procedures

#### 2.7.1. Ihh Signal Blocking by Cyclopamine

DMEM with 10 *µ*M cyclopamine was added into four 200 mL flasks (10 mL each) and incubated in a 37°C constant-temperature incubator with 5% CO_2_, for 3, 6, 12, or 48 h.

#### 2.7.2. PTHrP Intervention

DMEM with 10 nM PTHrP was added into four 200 mL flasks (10 mL each) and incubated in a 37°C constant-temperature incubator with 5% CO_2_, for 3, 6, 12, or 48 h.

#### 2.7.3. Ihh/PTHrP Double-Intervention Group

DMEM with 10 *µ*M cyclopamine + 10 nM PTHrP was added into four 200 mL flasks (10 mL each) and incubated in a 37°C constant-temperature incubator with 5% CO_2_, for 3, 6, 12, or 48 h.

#### 2.7.4. Control Group

DMEM with 10% FBS + 1% antibiotics was added into four 200 mL flasks (10 mL each) and incubated in a 37°C incubator with 5% CO_2_, for 3, 6, 12, or 48 h.

#### 2.7.5. Stop Reaction

The cultures were stopped at each time point by removing the culture media and washed twice with phosphate-buffered saline (PBS). The cells were collected for RT-PCR and Western blot. Detection of Ihh and PTHrP gene expression using RT-PCR and protein synthesis using Western blot. Image Pro-Plus was used to quantitative analyzed the result of Western blot.

#### 2.7.6. Flexecell Tension Plus System, FX-4000

This experiment used Flexecell Tension Plus system (FX-4000, Flexcellint) FX-4000 to provide tension during cells culturing. Experimental cells were cultured in Bioflex 6 holes' dishes, in which the bottom of the dish is silicone elastic membrane. Computer control of the vacuum pump makes silicone membrane produce tensile deformation at the bottom of the dish so that the bottom of the attached growth of cell indirectly accepts the tension. Cells are subjected to tension in the body and undergo specific biochemical changes to respond and adapt to deformation.

### 2.8. Statistics

The data were analyzed using SPSS 16.0 (SPSS, IL, USA). Each test was repeated three times under the same conditions to get average data as mean ± standard error of means. Statistical analysis of all parameters was performed using analysis of variance and Tukey test. A *P* value less than 0.05 was considered statistically significant.

## 3. Results

**Figure 1:**
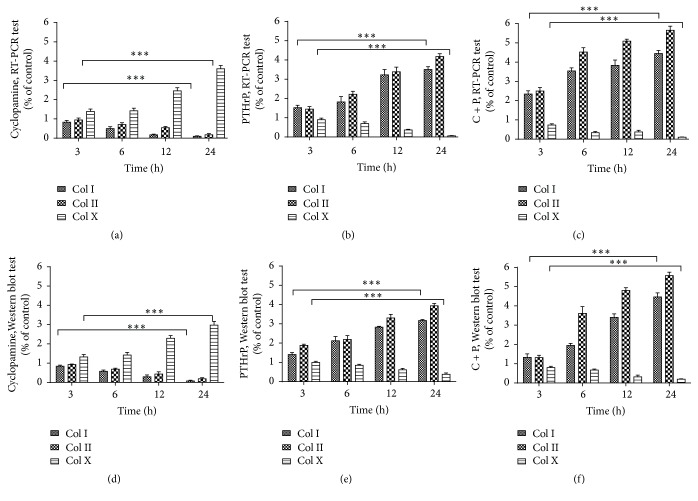
Col I, Col II, and Col X gene expression and protein synthesis under different intervention conditions. Note: (a), (b), and (c) RT-PCR results of Col I, Col II, and Col X gene expression levels by interventions with cyclopamine, PTHrP, and cyclopamine + PTHrP after 3, 6, 12, and 24 h. (d), (e), and (f) Western blot results of protein synthesis under the aforementioned conditions. All data were normalized to the control group (*n* = 3, ^*∗∗∗*^ *P* < 0.001, NS: nonsignificant).

**Figure 2:**
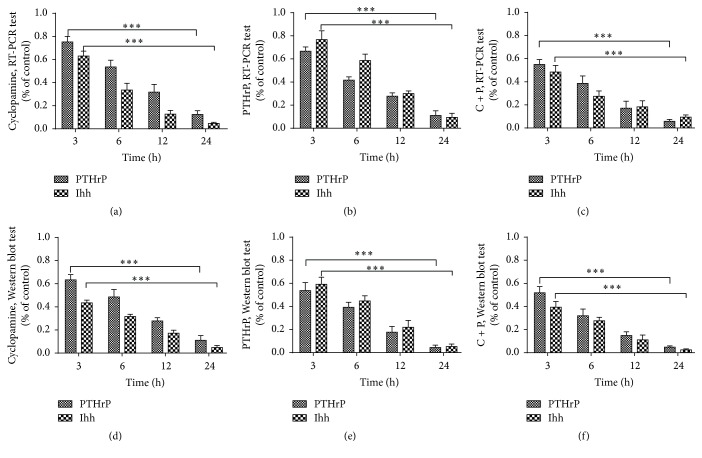
Ihh and PTHrP gene expression and protein synthesis under different intervention conditions. Note: (a), (b), and (c) RT-PCR results of Ihh and PTHrP gene expression levels by interventions with cyclopamine, PTHrP, and cyclopamine + PTHrP after 3, 6, 12, and 24 h. (d), (e), and (f) Western blot results of protein synthesis under the aforementioned conditions. All data were normalized to the control group (*n* = 3, ^*∗∗∗*^ *P* < 0.001, NS: nonsignificant).

After inhibition of Ihh signaling upstream of PTHrP by adding cyclopamine, PTHrP gene expression decreased significantly over time (*P* < 0.05) (Figure 2(a)). The relative values of PTHrP expression in the intervention groups versus the control group were 0.75 ± 0.08, 0.54 ± 0.10, 0.32 ± 0.12, and 0.12 ± 0.06 after 3, 6, 12, and 24 h, respectively (Table 3). Meanwhile, Col I and Col II expression decreased over time as well (*P* < 0.05) (Figure 1(a)). The relative values of Col I and Col II expression decreased from 0.83 ± 0.17 and 0.95 ± 0.15 to 0.10 ± 0.04 and 0.18 ± 0.10, from 3 to 24 h of signal inhibition, respectively. Col X expression, however, increased over time (*P* < 0.05) (Figure 1(a)). The relative gene expression of Col X increased from 1.39 ± 0.21 to 3.61 ± 0.29 (from 3 to 24 h of Ihh inhibition) (Table 1). Col I, Col II, and Col X protein synthesis under different intervention conditions was displayed in Table 2. Ihh and PTHrP protein synthesis under different intervention conditions was displayed in Table 4. Western blot of Col I, Col II, and Col X protein expression under different intervention conditions was displayed in Supplementary Figure 1 (in Supplementary Material available online at http://dx.doi.org/10.1155/2016/8235172). And RT-PCR of each gene expression under different intervention conditions was displayed in Supplementary Figures 2–6.

After adding PTHrP, PTHrP gene expression decreased significantly over time (*P* < 0.05) (Figure 2(b)). The relative gene expression levels at 3, 6, 12, and 24 h were 0.67 ± 0.06, 0.42 ± 0.04, 0.28 ± 0.05, and 0.11 ± 0.07, respectively (Table 3). Col I and Col II gene expression and protein synthesis increased significantly (*P* < 0.05) (Figure 1(b)). Col I and Col II gene expression levels increased from 1.52 ± 0.22 and 1.45 ± 0.22 at 3 h to 3.51 ± 0.25 and 4.18 ± 0.26 at 24 h. However, Col X expression decreased over time (*P* < 0.05) (Figure 1(b)), from 0.90 ± 0.15 at 3 h to 0.06 ± 0.04 at 24 h (Table 1).

In the Ihh/PTHrP double-intervention group, Col I and Col II gene expression and protein synthesis increased (*P* < 0.05) (Figure 1(b)) from 2.35 ± 0.29 and 2.50 ± 0.32 at 3 h to 4.45 ± 0.28 and 5.66 ± 0.36 at 24 h (Table 1).

In CTS experiments, using a 4%, 8%, 12% strain load at 1 Hz, types I, II, and X collagen mRNA expression and protein secretion increased with increased exposure time (*P* < 0.05) (Figure 4). Thus, results show that appropriate mechanical stimulation promoted tendon development by the secretion of types I and II collagen, while excessive mechanical stimulation increased the degeneration of tendon tissue into mineralized tissue.

**Figure 3:**
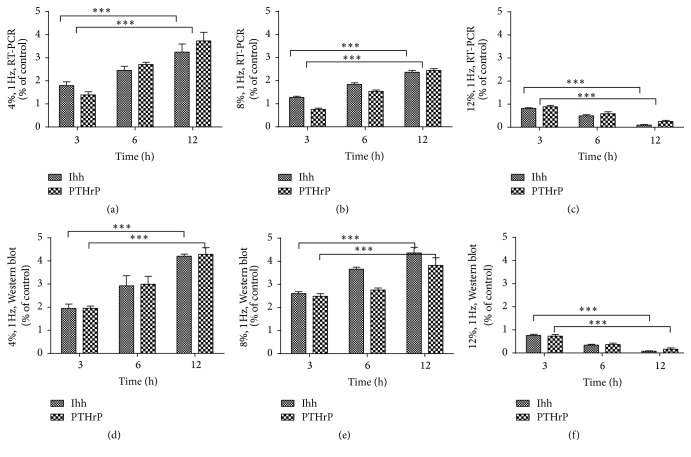
Ihh and PTHrP collagen mRNA expression under 4% (a), 8% (b), and 12% (c) CTS load for 3, 6, and 12 h. Ihh and PTHrP protein expression under 4% (d), 8% (e), and 12% (f) CTS load for 3, 6, and 12 h. Values are presented relative to the negative control group (without CTS, value of 1). All data were normalized to the control group (*n* = 3, ^*∗∗∗*^ *P* < 0.001, NS: nonsignificant).

**Figure 4:**
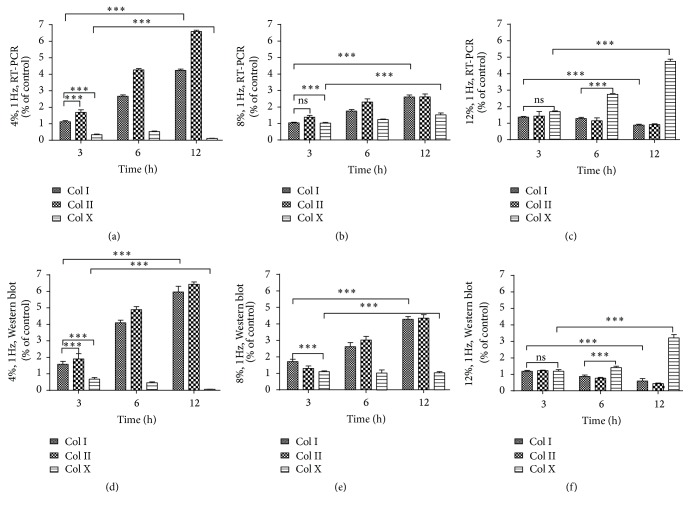
Types I, II, and X collagen mRNA expression under 4% (a), 8% (b), and 12% (c) CTS load for 3, 6, and 12 h. Types I, II, and X collagen protein expression under 4% (d), 8% (e), and 12% (f) CTS load for 3, 6, and 12 h. Values are presented relative to the negative control group (without CTS, value of 1). All data were normalized to the control group (*n* = 3, ^*∗∗∗*^ *P* < 0.001, ns: nonsignificant).

In CTS experiments, using a 4%, 8% strain load at 1 Hz, PTHrP and Ihh mRNA expression and protein secretion increased with increased duration of strain load (Figures 3(a), 3(b), 3(d), and 3(e)). But at 12% strain load at 1 Hz, PTHrP and Ihh mRNA expression and protein secretion reduced with increased time of strain load. These results suggest that mechanical stimulation affected PTHrP secretion.

## 4. Discussion

Chondrocyte proliferation and differentiation are controlled by a series of growth factors and endocrine hormones, of which Ihh/PTHrP signaling pathway plays the most important regulatory role [15–17]. During cartilaginous osteogenesis, the main role of PTHrP is to promote chondrocyte proliferation. Meanwhile, PTHrP inhibits chondrocyte hypertrophy, to inhibit chondrocyte differentiation and maturation. Ihh/PTHrP signaling pathway is an important but complex regulatory pathway to control cartilage development.

It has been proved that PTHrP stimulates chondrocyte proliferation but inhibits chondrocyte maturation, hence prolonging the cartilaginous osteogenesis, and helps in the formation of the complex skeleton shape and structure. Chondrocyte proliferation and differentiation were more rapid in PTHrP gene knockout mice than in wild-type mice. Also, the terminal differentiation of chondrocytes was faster, while the accretion zone at the cartilage growth plate shortened significantly [10]. In contrast, overexpression of PTHrP or continuous activation of parathyroid hormone 1 receptor resulted in the inhibition of chondrocyte differentiation and hypertrophy. PTHrP downregulates bone morphogenetic protein (BMP) signaling through RunX2, inhibiting osteoblast differentiation [18]. In addition, Col X is a marker of chondrocyte differentiation and maturation. PTHrP selectively inhibits Col X gene expression and protein synthesis, so as to inhibit chondrocyte differentiation and maturation [19, 20].

Ihh is known to be the most important cytokine to regulate chondrocyte proliferation and differentiation, playing a vital role during the cartilaginous osteogenesis [21, 22]. Ihh was found to be the primary upstream molecule of PTHrP, using the Ihh^−/−^ mice. In the absence of hedgehog protein, Ihh inhibits the activity of transmembrane protein Smoothened (Smo). With low levels of Ihh synthesis, hypertrophic chondrocytes at the growth plate expanded and chondrocyte proliferation decreased, resulting in dwarf malformation of the short limb. In the Ihh^−/−^ model, impaired chondrocyte proliferation and differentiation, or impaired osteoblast formation and mineralized bone structures, were noted, indicating that Ihh participates in regulating the cartilaginous osteogenesis [15, 16]. Besides, multiple bone defect diseases are related to Ihh gene mutation.

It was reported that when PTHrP was added, PTHrP and Ihh gene expression levels decreased significantly over time. Therefore, excess PTHrP inhibited Ihh expression and synthesis, resulting in decreased PTHrP expression. A study with the Ihh/PTHrP signaling pathway model proved that activation of Ihh signaling upregulates PTHrP expression. PTHrP increases the number of proliferating chondrocytes by inhibiting chondrocyte hypertrophy. At the same time, Ihh and PTHrP precisely regulate the growth of long bone through a feedback regulatory mechanism [23, 24].

The Ihh/PTHrP signaling pathway is as follows: hedgehog protein combines with Ihh receptor patched (Ptch) to stimulate a series of complex regulations. Transmembrane protein Smoothened (Smo) is activated, which directly induces transcriptional regulation of its target gene Ihh receptors [25], leading to increased PTHrP synthesis in chondrocytes and elevated PTHrP level around the chondrocytes, diffusing to the anterior hypertrophic chondrocyte region, promoting chondrocyte proliferation, and inhibiting chondrocyte differentiation. Van Donkelaar and Huiskes [9] proved that PTHrP-related factors are major regulatory molecules for chondrocyte hypertrophy, and Ihh-related factors are major regulatory molecules for chondrocyte proliferation. Ihh and PTHrP play different roles during chondrocyte proliferation and differentiation. Ihh regulates chondrocyte proliferation and differentiation during the resting stage through induction of its downstream signaling molecule PTHrP, thus maintaining the properties and functions of chondrocytes [26].

Ihh signaling pathway inhibitor cyclopamine is a plant steroidal alkaloid that inhibits Ihh signaling through antagonism with Smo [27, 28]. Some studies also indicate that cyclopamine may inhibit gene expression of Ihh pathway–related signaling molecules including PTHrP, Ihh, and Ptch [29]. The downregulation of target genes is dose dependent. Therefore, cyclopamine inhibits Ihh signaling pathway effectively. According to studies by Mak et al. [30], Shimoyama et al. [31], and Mak et al. [32], 10 *µ*M cyclopamine, which is the half-effective inhibition concentration, evidently deferred chondrocyte hypertrophy and proliferation, indicating that PTHrP might induce fibrochondrocyte proliferation and inhibit its maturation. In this experiment, cyclopamine blocked PTHrP upstream signaling molecule Ihh and decreased PTHrP gene expression over time. Therefore, this study proved that PTHrP expression is affected by its upstream signaling molecule Ihh.

Fibrochondrocytes specifically synthesize Col I and Col II. It was shown that Col I and Col II were replaced by Col X along with the differentiation of chondrocyte toward hypertrophic fibrous chondrocytes, and acidic glycosaminoglycan secretion decreased within the mesochondrium. Meanwhile, cell activities decreased. Therefore, Col X was considered to be an important parameter for assessing fibrochondrocyte proliferation and differentiation [33]. Col X is used to evaluate the degree of fibrochondrocyte differentiation. The results showed that PTHrP evidently promoted Col I and Col II gene expression and protein synthesis and inhibited Col X gene expression and protein synthesis, indicating that PTHrP may induce fibrochondrocyte proliferation and inhibit its maturation. When its upstream Ihh signaling pathway was blocked by cyclopamine, PTHrP gene expression and protein synthesis both decreased. In contrast, Col X gene expression and protein synthesis both increased, indicating that Ihh promotes fibrochondrocyte maturation and hypertrophy in entheses. In the Ihh/PTHrP double-intervention group, Col I and Col II gene expression and protein synthesis increased. Therefore, PTHrP might induce Col I and Col II gene expression and protein synthesis to promote chondrocyte proliferation. This effect was not affected by Ihh signal block. Ihh induced PTHrP expression, and PTHrP overexpression downregulated Ihh. PTHrP and Ihh coordinately regulated chondrocyte proliferation and differentiation in entheses through a negative feedback mechanism. This proved that Ihh signal regulates fibrochondrocyte differentiation in entheses through PTHrP.

In summary, Ihh/PTHrP signaling pathway is the crucial regulator in regulating fibrochondrocyte development in entheses. Ihh is synthesized by anterior hypertrophic chondrocytes of the fibrous cartilaginous layer in entheses. By autocrine and paracrine secretion, Ihh combines with transmembrane Ihh receptors on fibrochondrocytes during the G0/G1 stage within the surface layer of fibrous cartilaginous layer to regulate and induce fibrochondrocyte proliferation and differentiation and increase PTHrP synthesis. PTHrP inhibits hypertrophic differentiation of chondrocytes within the fibrous cartilaginous zone of entheses. Meanwhile, PTHrP inhibits Ihh synthesis in anterior hypertrophic chondrocytes in the fibrous cartilaginous layer.

Ihh/PTHrP signaling pathway is an important regulator for fibrochondrocyte proliferation and differentiation in entheses. Ihh signal controls fibrochondrocyte differentiation in entheses through PTHrP to promote fibrochondrocyte hypertrophy and maturation, while PTHrP induces fibrochondrocyte proliferation but inhibits the differentiation and maturation. Ihh and PTHrP signals coexist in entheses, forming a signaling pathway to interactively regulate fibrochondrocyte proliferation and differentiation through a negative feedback loop, ensuring the physiological process of entochondrostosis, maintaining the balance of the fibrous cartilaginous layer growth and differentiation and thereby protecting the physiological functions of entheses.

Our study has limitations. Despite the fact that FX-4000 system can simulate the investigating cellular responses to mechanical loads with a maximum ability, it is still unable to simulate the mechanical loads of the Entheses of pig Achilles tendon during different motion.

## 5. Conclusions

In this study, Ihh and PTHrP coregulate fibrochondrocyte proliferation and differentiation in entheses through negative feedback regulation. Low tensile strength of CTS (4%, 3 h, 1 Hz) promotes cell proliferation, and high tensile strength of CTS (12%, 12 h, 1 Hz) causes differentiation of fibrochondrocytes. This phenomenon is regulated by stress stimulation through the Ihh/PTHrP signaling pathway.

## Supplementary Material

Supplementary Material: Western blot of Col I, Col II and Col X protein expressions and RT-PCR of Col I, Col II, Col X, PTHrP and Ihh gene expressions under different intervention conditions.

## Figures and Tables

**Table 1:** Col I, Col II, and Col X gene expression results under different intervention conditions (*n* = 3, $\overset{-}{x}\pm s$).

	Collagen type I				Collagen type II				Collagen type X			
	3 h	6 h	12 h	24 h	3 h	6 h	12 h	24 h	3 h	6 h	12 h	24 h
Cyclo	0.83 ± 0.17	0.50 ± 0.17	0.17 ± 0.07	0.10 ± 0.04	0.95 ± 0.15	0.71 ± 0.18	0.54 ± 0.11	0.18 ± 0.10	1.39 ± 0.21	1.42 ± 0.23	2.46 ± 0.29	3.61 ± 0.29
PTHrP	1.52 ± 0.22	1.82 ± 0.47	3.23 ± 0.48	3.51 ± 0.25	1.45 ± 0.22	2.22 ± 0.27	3.39 ± 0.42	4.18 ± 0.26	0.90 ± 0.15	0.70 ± 0.16	0.37 ± 0.06	0.06 ± 0.04
C + P	2.35 ± 0.29	3.54 ± 0.28	3.83 ± 0.48	4.45 ± 0.28	2.50 ± 0.32	4.53 ± 0.39	5.09 ± 0.18	5.66 ± 0.36	0.74 ± 0.12	0.34 ± 0.11	0.38 ± 0.15	0.10 ± 0.02

Note: cyclo: Ihh block by cyclopamine; C + P: double intervention by cyclopamine + PTHrP; *n* = 3, 3 h versus 24 h comparison.

**Table 2:** Col I, Col II, and Col X protein synthesis under different intervention conditions (*n* = 3, $\overset{-}{x}\pm s$).

	Collagen type I				Collagen type II				Collagen type X			
	3 h^*∗*^	6 h	12 h	24 h^*∗*^	3 h^*∗*^	6 h	12 h	24 h^*∗*^	3 h^*∗*^	6 h	12 h	24 h^*∗*^
Cyclo	0.84 ± 0.10	0.58 ± 0.11	0.29 ± 0.17	0.08 ± 0.06	0.93 ± 0.04	0.69 ± 0.09	0.43 ± 0.21	0.19 ± 0.10	1.32 ± 0.22	1.42 ± 0.24	2.29 ± 0.23	2.97 ± 0.34
PTHrP	1.41 ± 0.18	2.12 ± 0.38	2.83 ± 0.07	3.17 ± 0.09	1.88 ± 0.10	2.19 ± 0.34	3.31 ± 0.32	3.94 ± 0.21	0.90 ± 0.11	0.85 ± 0.10	0.62 ± 0.13	0.38 ± 0.12
C + P	1.33 ± 0.32	1.95 ± 0.19	3.41 ± 0.32	4.47 ± 0.37	1.32 ± 0.21	3.61 ± 0.60	4.80 ± 0.25	5.57 ± 0.32	0.80 ± 0.12	0.67 ± 0.09	0.32 ± 0.16	0.20 ± 0.04

Note: cyclo: Ihh block by cyclopamine; C + P: double intervention by cyclopamine + PTHrP; *n* = 3, 3 h versus 24 h comparison: ^*∗*^ *P* < 0.01.

**Table 3:** Ihh and PTHrP gene expression under different intervention conditions (*n* = 3, $\overset{-}{x}\pm s$).

	Ihh				PTHrP			
	3 h^*∗*^	6 h	12 h	24 h^*∗*^	3 h^*∗*^	6 h	12 h	24 h^*∗*^
Cyclo	0.63 ± 0.07	0.34 ± 0.10	0.13 ± 0.06	0.05 ± 0.02	0.75 ± 0.08	0.54 ± 0.10	0.32 ± 0.12	0.12 ± 0.06
PTHrP	0.7 ± 0.13	0.59 ± 0.10	0.30 ± 0.04	0.10 ± 0.06	0.67 ± 0.06	0.42 ± 0.05	0.28 ± 0.05	0.11 ± 0.07
C + P	0.48 ± 0.10	0.27 ± 0.08	0.18 ± 0.09	0.09 ± 0.03	0.55 ± 0.07	0.39 ± 0.11	0.17 ± 0.11	0.06 ± 0.03

Note: cyclo: Ihh block by cyclopamine; C + P: double intervention by cyclopamine + PTHrP; *n* = 3, 3 h versus 24 h comparison: ^*∗*^ *P* < 0.01.

**Table 4:** Ihh and PTHrP protein synthesis under different intervention conditions (*n* = 3, $\overset{-}{x}\pm s$).

	Ihh				PTHrP			
	3 h^*∗*^	6 h	12 h	24 h^*∗*^	3 h^*∗*^	6 h	12 h	24 h^*∗*^
Cyclo	0.43 ± 0.04	0.32 ± 0.04	0.17 ± 0.05	0.05 ± 0.03	0.63 ± 0.08	0.49 ± 0.11	0.28 ± 0.05	0.11 ± 0.07
PTHrP	0.59 ± 0.10	0.45 ± 0.08	0.22 ± 0.10	0.05 ± 0.04	0.54 ± 0.12	0.39 ± 0.08	0.18 ± 0.09	0.04 ± 0.04
C + P	0.39 ± 0.09	0.28 ± 0.05	0.11 ± 0.07	0.02 ± 0.01	0.52 ± 0.09	0.32 ± 0.1	0.15 ± 0.06	0.05 ± 0.02

Note: cyclo: Ihh block by cyclopamine; C + P: double intervention by cyclopamine + PTHrP; *n* = 3, 3 h versus 24 h comparison: ^*∗*^ *P* < 0.01.
