# Supplementary material for: Regulatory Mechanisms of the Ihh/PTHrP Signaling Pathway in Fibrochondrocytes in Entheses of Pig Achilles Tendon
Source: Stem Cells Int. 2016 Nov 22;2016:8235172. doi: 10.1155/2016/8235172 (PMC5138489; doi:10.1155/2016/8235172)
Supplement: Supplementary file 1 — Supplementary Material: Western blot of Col I, Col II and Col X protein expressions and RT-PCR of Col I, Col II, Col X, PTHrP and Ihh gene expressions under different intervention conditions. [file 8235172.f1.zip › Supplementary Material/Supplementary figure 3.pdf]

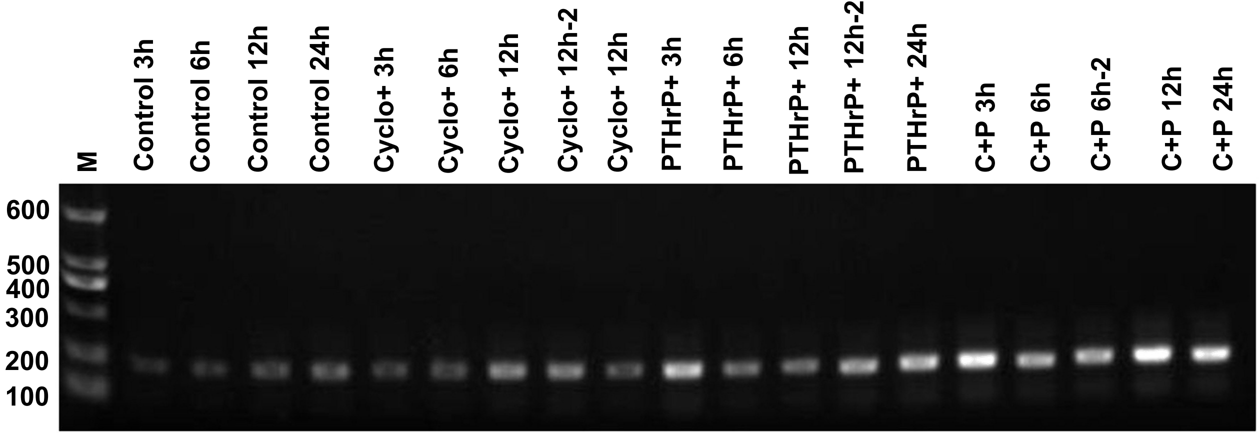

M
Control 3h
Control 6h
Control 12h
Control 24h
Cyclo+ 3h
Cyclo+ 6h
Cyclo+ 12h
Cyclo+ 12h-2
Cyclo+ 12h
PTHrP+ 3h
PTHrP+ 6h
PTHrP+ 12h
PTHrP+ 12h-2
PTHrP+ 24h
C+P 3h
C+P 6h
C+P 6h-2
C+P 12h
C+P 24h
600
500
400
300
200
100
