# Supplementary material for: Regulatory Mechanisms of the Ihh/PTHrP Signaling Pathway in Fibrochondrocytes in Entheses of Pig Achilles Tendon
Source: Stem Cells Int. 2016 Nov 22;2016:8235172. doi: 10.1155/2016/8235172 (PMC5138489; doi:10.1155/2016/8235172)
Supplement: Supplementary file 1 — Supplementary Material: Western blot of Col I, Col II and Col X protein expressions and RT-PCR of Col I, Col II, Col X, PTHrP and Ihh gene expressions under different intervention conditions. [file 8235172.f1.zip › Supplementary Material/Supplementary figure legends.docx]

**Supplementary Figures**

**Supplementary figure 1.** Western blot of Col I, Col II, and Col X protein expression under different intervention conditions.

**Supplementary figure 2.** RT-PCR of Col I gene expression under different intervention conditions.

*Note*: Cyclo: Ihh block by cyclopamine; PTHrP: enhancement of PTHrP; C + P: double intervention by cyclopamine + PTHrP.

**Supplementary figure 3.** RT-PCR of Col II gene expression under different intervention conditions.

*Note*: Cyclo: Ihh block by cyclopamine; PTHrP: enhancement of PTHrP; C + P: double intervention by cyclopamine + PTHrP.

**Supplementary figure 4.** RT-PCR of Col X gene expression under different intervention conditions.

*Note*: Cyclo: Ihh block by cyclopamine; PTHrP: enhancement of PTHrP; C + P: double intervention by cyclopamine + PTHrP.

**Supplementary figure 5.** RT-PCR of PTHrP gene expression under different intervention conditions.

*Note*: Cyclo: Ihh block by cyclopamine; PTHrP: enhancement of PTHrP; C + P: double intervention by cyclopamine + PTHrP.

**Supplementary figure 6.** RT-PCR of Ihh gene expression under different intervention conditions

*Note*: Cyclo: Ihh block by cyclopamine; PTHrP: enhancement of PTHrP; C + P: double intervention by cyclopamine + PTHrP.
