# Supplementary figures and images for: Regulatory Mechanisms of the Ihh/PTHrP Signaling Pathway in Fibrochondrocytes in Entheses of Pig Achilles Tendon
Source: Stem Cells Int. 2016 Nov 22;2016:8235172. doi: 10.1155/2016/8235172 (PMC5138489; doi:10.1155/2016/8235172)

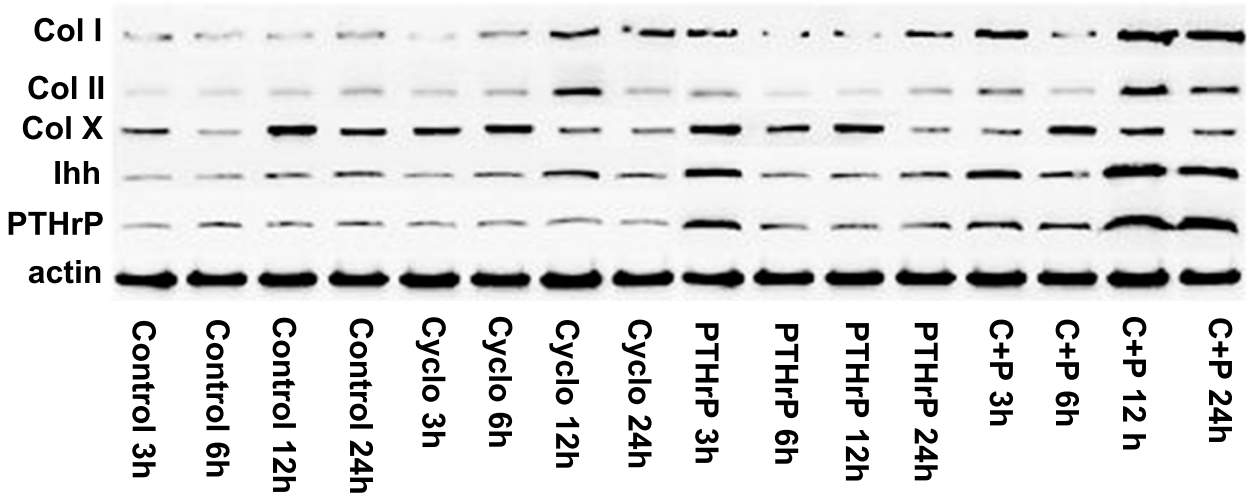

Supplement: Supplementary file 1 — Supplementary Material: Western blot of Col I, Col II and Col X protein expressions and RT-PCR of Col I, Col II, Col X, PTHrP and Ihh gene expressions under different intervention conditions. [file 8235172.f1.zip › Supplementary Material/Supplementary figure 1.pdf]

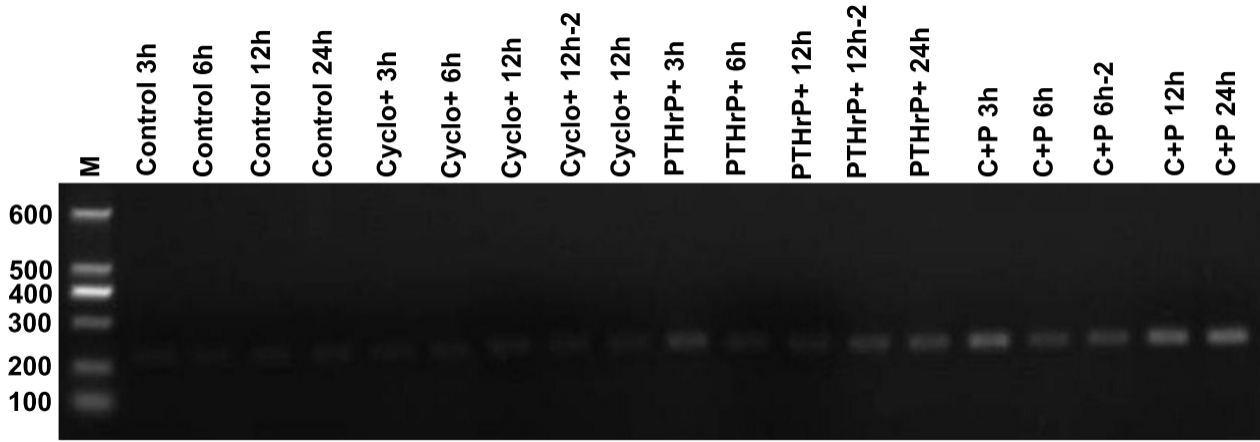

Supplement: Supplementary file 1 — Supplementary Material: Western blot of Col I, Col II and Col X protein expressions and RT-PCR of Col I, Col II, Col X, PTHrP and Ihh gene expressions under different intervention conditions. [file 8235172.f1.zip › Supplementary Material/Supplementary figure 2.pdf]

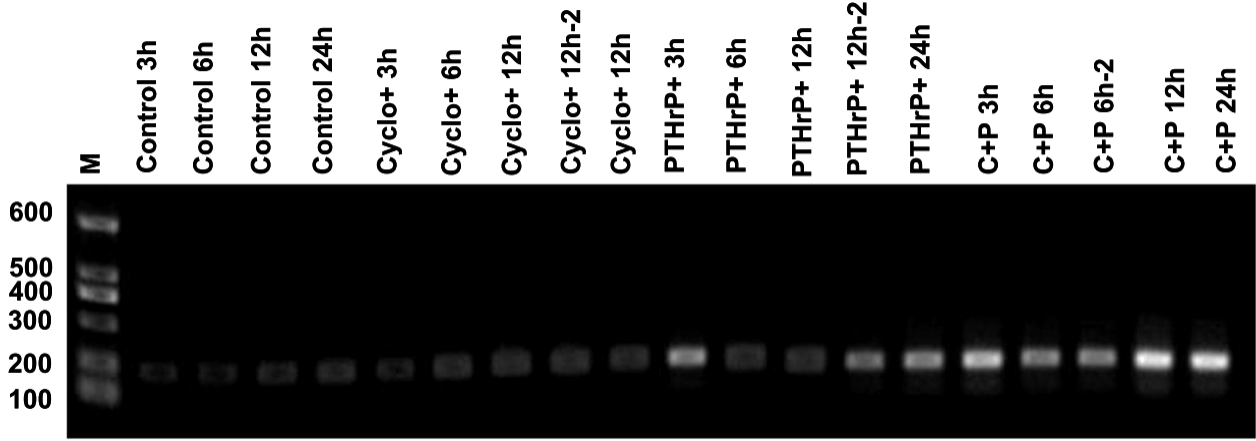

Supplement: Supplementary file 1 — Supplementary Material: Western blot of Col I, Col II and Col X protein expressions and RT-PCR of Col I, Col II, Col X, PTHrP and Ihh gene expressions under different intervention conditions. [file 8235172.f1.zip › Supplementary Material/Supplementary figure 5.pdf]

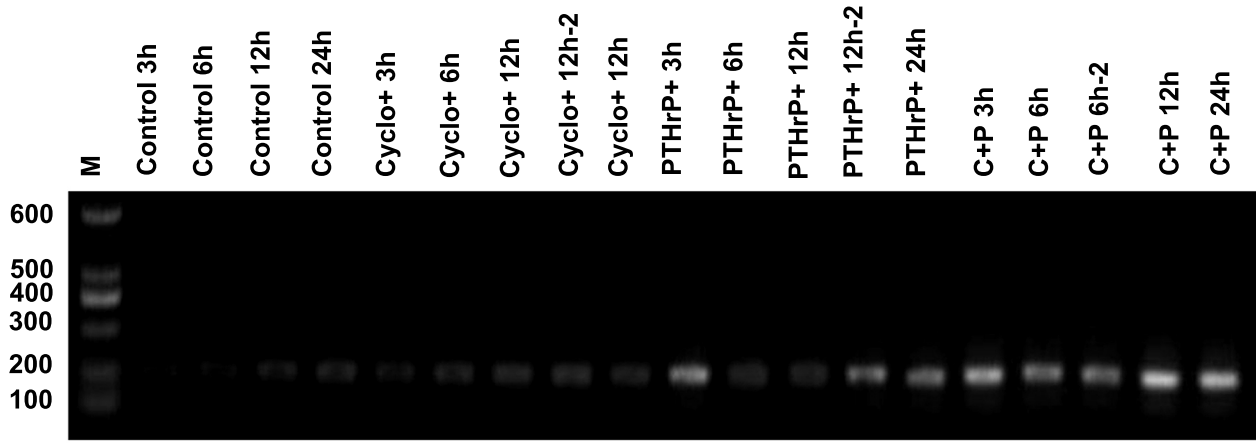

Supplement: Supplementary file 1 — Supplementary Material: Western blot of Col I, Col II and Col X protein expressions and RT-PCR of Col I, Col II, Col X, PTHrP and Ihh gene expressions under different intervention conditions. [file 8235172.f1.zip › Supplementary Material/Supplementary figure 6.pdf]
